# Fulvestrant inhibits growth of triple negative breast cancer and synergizes with tamoxifen in ERα positive breast cancer by up-regulation of ERβ

**DOI:** 10.18632/oncotarget.10871

**Published:** 2016-07-28

**Authors:** Ameet K. Mishra, Annelie Abrahamsson, Charlotta Dabrosin

**Affiliations:** ^1^ Department of Oncology and Department of Clinical and Experimental Medicine, Linköping University, Linköping, Sweden

**Keywords:** mammary cancer, sex steroids, steroid receptors, proliferation, DNA methyltransferase

## Abstract

The estrogen receptor-alpha (ERα) is used as a predictive marker for anti-estrogen therapy in breast cancer patients. In addition to aromatase inhibitors, ERα can be targeted at the receptor level using the receptor modulator tamoxifen or by the pure anti-estrogen fulvestrant. The role of the second ER, ER-beta (ERβ), as a therapeutic target or prognostic marker in breast cancer is still elusive. Hitherto, it is not known if ERα+/ERβ+ breast cancers would benefit from a treatment strategy combining tamoxifen and fulvestrant or if fulvestrant exert any therapeutic effects in ERα-/ERβ+ breast cancer. Here, we report that fulvestrant up-regulated ERβ in ERα+/ERβ+ breast cancer and in triple negative ERβ+ breast cancers (ERα-/ERβ+). In ERα+/ERβ+ breast cancer, a combination therapy of tamoxifen and fulvestrant significantly reduced tumor growth compared to either treatment alone both *in vivo* and *in vitro*. In ERα-/ERβ+ breast cancer fulvestrant had potent effects on cancer growth, *in vivo* as well as *in vitro*, and this effect was dependent on intrinsically expressed levels of ERβ. The role of ERβ was further confirmed in cells where ERβ was knocked-in or knocked-down. Inhibition of DNA methyltransferase (DNMT) increased the levels of ERβ and fulvestrant exerted similar potency on DNMT activity as the DNMT inhibitor decitabine. We conclude that fulvestrant may have therapeutic potential in additional groups of breast cancer patients; i) in ERα+/ERβ+ breast cancer where fulvestrant synergizes with tamoxifen and ii) in triple negative/ERβ+ breast cancer patients, a subgroup of breast cancer patients with poor prognosis.

## INTRODUCTION

Estrogen exposure is implicated in the development and progression of breast cancer and approximately two thirds of all breast cancers express estrogen receptor alpha (ERα) [1]. ERα is a predictive marker for anti-estrogen therapy but only approximately 50% of the patients will benefit from anti-estrogen therapy using this marker [1]. ERα can be targeted using at least three different approaches such as modulation of the receptor by selective estrogen receptor modulators (SERMs), down-regulation of the receptor by estrogen receptor down regulators (SERDs) or by decreasing the production of estradiol by aromatase inhibitors (AIs) [2]. The SERM tamoxifen, is one of the most effective and widely prescribed drugs for breast cancer [3]. The mechanism of action of tamoxifen is complex and its effects may be agonistic or antagonistic depending on the target tissue. In the breast, tamoxifen is considered an antagonistic by its binding to ERα and thereby blocking the proliferative actions of estrogen [4]. The SERD fulvestrant, ICI 182,780, on the other hand, acts as a pure anti-estrogen by hindering receptor dimerization, increasing receptor turnover, and disrupting the nuclear localization of ERα [5, 6]. This is a marked contrast to the stable or even increased ERα expression caused by tamoxifen [5]. Fulvestrant has been shown to be equally effective as AI in postmenopausal women with advanced breast cancer and no cross-resistance between the drugs has been detected [7]. Combining the different routes of action on the ERα signaling would theoretically improve patient outcome but in the ATAC trial the combination arm of anastrozol (an AI) in combination with tamoxifen was discontinued because of a lack of improved efficacy compared to anastrozol alone [8]. In the metastatic setting the FACT study, fulvestrant in combination with anastrozol, did not offer any advantage compared to anastrozol alone [9]. In these trials ERα was determined and targeted. A second type of ER, estrogen receptor beta (ERβ) was discovered two decades ago [10]. ERβ is encoded by a gene on chromosome 14 and has 56% similarity in ligand-binding domain with ERα [11–15]. Divergent function of ERα and ERβ has been suggested in breast cancer. ERβ is predominant in normal breast tissues while ERα is expressed only in 10-20% of breast epithelial cells [16]. During breast carcinogenesis ERβ expression is gradually lost and ERα becomes the dominant subtype of ER [17–20]. Previous studies suggest that fulvestrant may stabilize or even increase ERβ expression suggesting that ERβ may be a target for fulvestrant [21, 22]. Considering ERα, a combination with fulvestrant and tamoxifen would not lead to any benefit compared to either treatment alone. However, it is not known if ERα+/ERβ+ expressing breast cancer would benefit using a treatment strategy combining tamoxifen and fulvestrant or if fulvestrant exert any therapeutic effects in ERα-/ERβ+ breast cancer. ERα-/ERβ+ breast cancers belong to the triple-negative breast cancer (TNBC) group, a subgroup of breast cancers characterized by the lack of expression of ERα, progesterone receptor, and human epidermal growth factor receptor-2 (HER-2) [23]. Approximately 10-15% of all breast cancers are TNBC and treatment options for TNBC are limited due to the absence of well-defined molecular targets [24–26]. In clinical materials it has been demonstrated that ERβ may be expressed in approximately 30% of all triple negative breast cancers [27]. ERβ may therefore be a potential target in TNBC but this has previously not been fully explored.

Here, we show that fulvestrant has potent therapeutic effects in ERα-/ERβ+ breast cancer and that tamoxifen and fulvestrant in combination enhance tumor regression of ERα+/ERβ+ breast cancer by up-regulation of ERβ.

## RESULTS

### Fulvestrant in combination with tamoxifen enhanced tumor regression *in vivo* compared with either treatment alone

**Figure 1:**
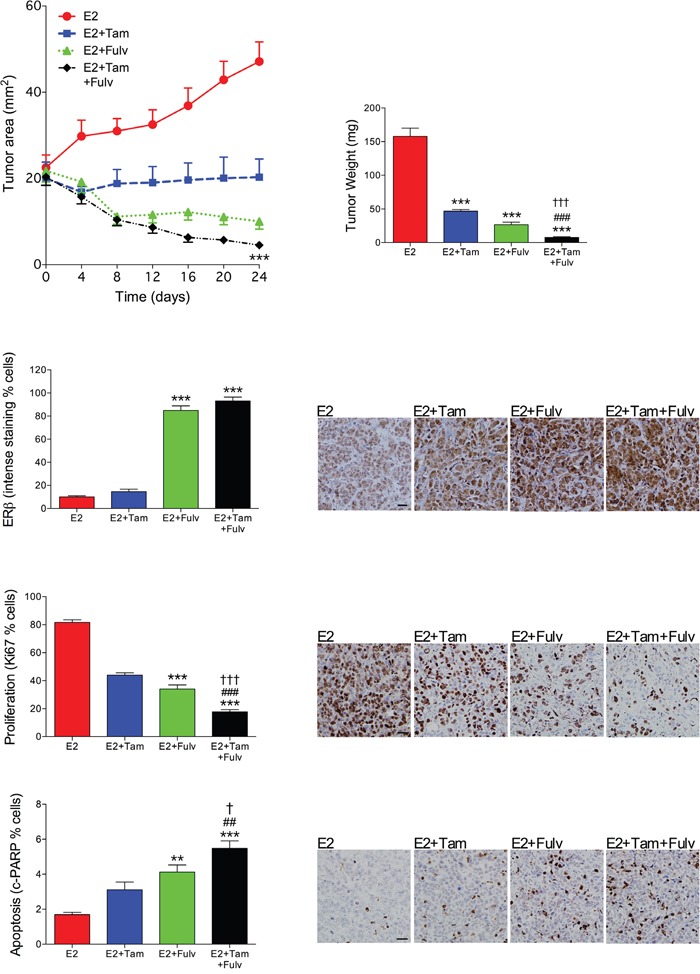
Fulvestrant in combination with tamoxifen enhanced tumor regression *in vivo* compared with either treatment alone OophorectomizedBalb/C-nu/nu mice were supplemented with physiological levels of estradiol (E2) and injected with MCF-7 cells in the mammary fat pad. At similar tumor sizes, one group continued with E2 treatment and the other group received an additional tamoxifen (Tam) treatment (1 mg/mouse every second day s.c.), fulvestant (Fulv) (5mg/mouse twice weekly s.c.), or their combination. Tumor sections from the different treatment groups were stained for ERβ (clone PPG5/10), proliferation (Ki67) or apoptosis (cleaved PARP (cPARP)) and quantified as described in Materials and Methods. Representative sections from each treatment group are shown. Scale bars=50 μm. **P<0.01 and ***P<0.001 compared to E2, ##P<0.01 and ###P<0.001 compared to E2+Tam, and † P<0.05 and ††† P<0.001 compared to E2+Fulv, n=8-21 in each group. Bars and dots represent mean±SEM.

To test if tumor regression of ERα+/ERβ+ breast cancer could be enhanced by a combination treatment of fulvestrant and tamoxifen, MCF-7 breast cancer explants were established in nude mice. As MCF-7 cells require estradiol for tumor growth in nude mice the various treatments were added with a stable background of estradiol at physiologic levels. At similar tumor sizes treatment with tamoxifen, fulvestrant or their combination was initiated. Fulvestrant treatment resulted in significantly decreased tumor growth compared to tamoxifen, Figure 1. The combination of fulvestrant and tamoxifen treatments resulted in significantly decreased tumor growth compared with either treatment alone, Figure 1. As fulvestrant in previous studies has been shown to affect ERβ expression the tumors from the different treatment groups were stained for ERβ. We found that fulvestrant increased ERβ expression in tumors treated with fulvestrant alone or in combination with tamoxifen, whereas tamoxifen alone did not affect ERβ compared with estrogen exposed tumors, Figure 1. In the combination group significant decreased proliferation (Ki67) was detected as well as increased apoptosis (cleaved PARP) compared with either treatment alone, Figure 1.

As expected, fulvestrant decreased ERα expression whereas tamoxifen increased the expression determined by % stained cells measured using immunohistochemistry; 47±7% in E2, 84±4% in E2+Tam, 18±2% in E2+Fulv, and 65±11% in E2+Tam+Fulv, n=8 in each group. Thus, fulvestrant down-regulated ERα by 60% whereas ERβ was up-regulated over seven times within the same tumors.

### Fulvestrant in combination with tamoxifen affected cell proliferation and ERβ expression *in vitro*

**Figure 2:**
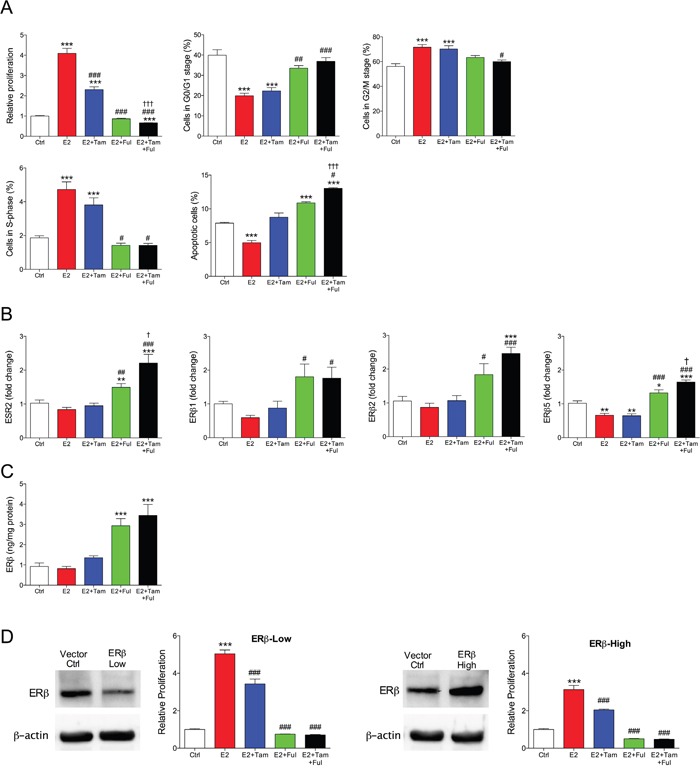
Fulvestrant in combination with tamoxifen affected cell proliferation and ERβ expression *in vitro* Cell culture of MCF-7 cells exposed to estradiol (E2) 10^−9^ M, tamoxifen (Tam) 10^−6^ M, fulvestrant (Fulv) 10^−6^ M, or their combination. **A.** Cell proliferation, cell cycle, apoptosis and cell cellular senescence analyses were performed. ***P<0.001 compared to control, #P<0.05 ##P<0.01 and ###P<0.001 compared to E2, and †P<0.05 and ††† P<0.001 compared to E2+Fulv, n=6-8 in each group. Bars represent mean±SEM. The control group represents the normalized absorbance of unexposed (hormone media alone) MCF-7 wild type cells. **B.** Total ERβ (ESR2) and ERβ isoforms (ERβ1, ERβ2, and ERβ5) levels were analyzed at the mRNA level in cultured MCF-7 cells. *P<0.05, **P<0.01 and ***P<0.001 compared to control, #P<0.05 ##P<0.01 and ###P<0.001 compared to E2, and †P<0.05 compared to E2+Fulv, n=6-8 in each group. Bars represent mean±SEM. **C.** Protein levels of ERβ measured with ELISA in MCF-7 cells exposed to hormones as described above. **D.** Parental MCF-7 cells were stable transfected with ESR2 shRNA or ESR2 vector to generate MCF-7/ERβ-Low and MCF-7/ERβ-High cells respectively. Western blot confirmed the transfections at protein levels. Cells were exposed to hormones as above. Cell proliferation analysis was performed. ***P<0.001 compared to control and ###P<0.001 compared to E2, n=6 in each group. Bars represent mean±SEM. The control group represents the normalized absorbance of unexposed (hormone media alone) MCF-7 cells treated with vehicle control (mock transfected) of each experiment.

To further delineate the effects of the treatments, MCF-7 cells were cultured *in vitro*. As expected, estradiol increased proliferation of MCF-7 cells whereas tamoxifen, fulvestrant or their combination counteracted the effects of estradiol, Figure 2A. Fulvestrant was more effective than tamoxifen and the combination of fulvestrant and tamoxifen decreased the proliferation rate significantly compared with fulvestrant alone, Figure 2A. Cell cycle analysis revealed that estradiol induced increase in cell division, which was restored to control levels after fulvestrant and combination treatment. There was a significant increase of apoptosis in the combination group compared with fulvestrant alone, Figure 2A.

### Fulvestrant increased ERβ expression of MCF-7 cells

In line with the *in vivo* data of increased ERβ protein by fulvestrant exposure, fulvestrant increased the expression of ERβ and its isoforms at the mRNA and protein levels, Figure 2B-2C. In addition, the combination of fulvestrant with tamoxifen increased the expression of ERβ and the isoforms ERβ2 and ERβ5 compared to fulvestrant alone, Figure 2B.

### Increased ERβ expression decreased cell proliferation

To elucidate the role of ERβ expression on cell proliferation of MCF-7 cells vectors were used to generate stable ERβ over-expression (MCF-7/ERβ-High), which resulted in a 1.3±0.03 fold increased of the expression, or ESR2 shRNA for a decrease of ERβ expression (MCF-7/ERβ-Low) resulting in a 0.5±0.01 fold decreased expression. This was also confirmed at protein levels, Figure 2D. In ERβ-high cells, the estradiol effects on cell proliferation was decreased while the inhibitory effect of fulvestrant on cell proliferation was increased, Figure 2C. Down-regulation of ERβ resulted in decreased inhibitory effects on cell proliferation by tamoxifen, fulvestrant, and their combination, Figure 2C. Treating MCF-7 cells with the selective ERβ antagonist PHTPP (4-[2-Phenyl-5,7-*bis*(trifluoromethyl)pyrazolo[1,5-*a*]pyrimidin-3-yl]phenol) also resulted in a significant increase in the proliferation *per se*, from 1±0.05 in untreated cells to 1.5±0.06 in the exposed cells, p<0.001, n=6 in each group. These results confirmed the role of ERβ in the regulation of proliferation in MCF-7 cells.

### Therapeutic effects of fulvestrant in triple negative ERβ+ MDA-MB-231 breast cancer explants

**Figure 3:**
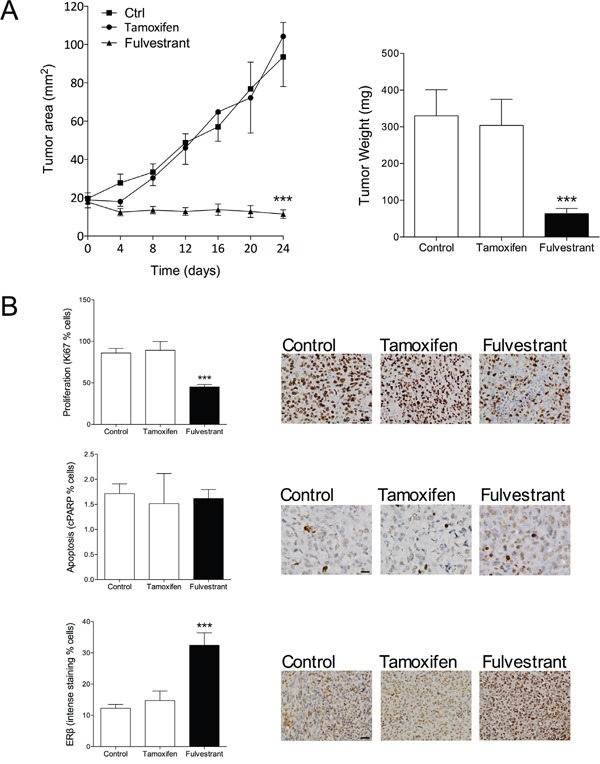
Therapeutic effect of fulvestrant in triple negative ERβ+ MDA-MB-231 breast cancer explants **A.** MDA-MB-231 cells (ERα-/ERβ+) were injected in the mammary fat pad in nude mice as described under figure 1. At similar tumor sizes, one group received tamoxifen treatment (1 mg/mouse every second day s.c.) or fulvestant (5 mg/mouse twice weekly s.c.). ***P<0.0001 compared to controls and tamoxifen, n=6-10 in each group. Dots represent mean±SEM. **B.** Tumor sections from the different treatment groups were stained for ERβ (clone PPG5/10), proliferation (Ki67) or apoptosis (cleaved PARP (cPARP)) and quantified as described in Materials and Methods. Representative sections from each treatment group are shown. Scale bars=50 μm. ***P<0.0001 compared to controls and tamoxifen, n=9 in each group. Bars represent mean±SEM.

**Figure 4:**
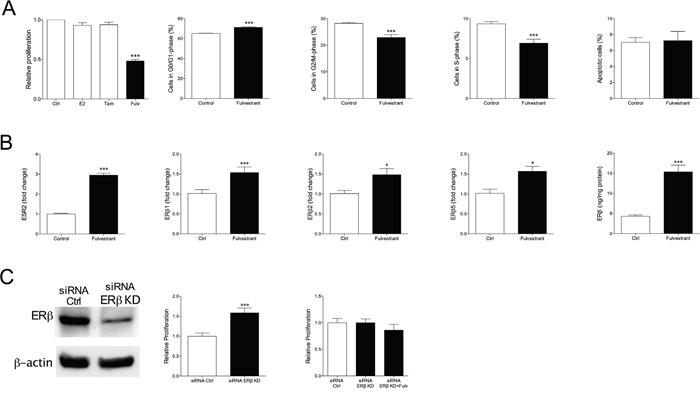
Culture of ERα-/ERβ+ MDA-MB-231 cells exposed to tamoxifen (Tam) 10-6 M or fulvestrant (Fulv) 10-6 M for 7 days **A.** Cell proliferation and cell cycle analyses performed. ***P<0.001 compared to control, n=6-10 in each group. Bars represent mean±SEM. **B.** ERβ and its isoforms were analyzed at mRNA levels and total ERβ protein was measured by ELISA, *P<0.05 and ***P<0.001 compared to control, n=6 in each group. Bars represent mean±SEM. **C.** The effects of fulvestrant in MDA-MB-231 cells were dependent on ERβ expression. ERβ was transiently knocked-down (KD) in MDA-MB-231 cells as described in the materials and methods section and the KD was confirmed at the protein level by Western blot. Cell proliferation was determined, ***P<0.001 compared to control, n=10 in each group. Bars represent mean±SEM.

Encouraged by our results in ERα+/ERβ+ breast cancer we investigated if fulvestrant had the ability to affect growth of ERα-/ERβ+ breast cancer. The MDA-MB-231 cells do not express ERα and ERβ is expressed at moderate levels. MDA-MB-231 cancer were established in the mammary fat pad in nude mice and treated with fulvestrant or tamoxifen. As expected, and previously shown [28], tamoxifen did not affect tumor growth, Figure 3A. Fulvestrant, however, significantly decreased tumor growth, Figure 3A. Staining of tumor sections revealed a significant decrease of proliferation by fulvestrant whereas no effects on apoptosis was detected, Figure 3B. Fulvestrant significantly increased the expression of ERβ in these tumors, Figure 3B. To delineate the results on a cellular level MDA-MB-231 cells were cultured *in vitro*. Cell cycle analyses confirmed the *in vivo* results and showed that fulvestrant decreased proliferation whereas no effects were seen on apoptosis, Figure 4A. Similar to the effects of fulvestrant on MCF-7 cells (ERα+/ERβ+) the mRNA levels of ERβ and its isoforms increased in MDA-MB-231 (ERα-/ERβ+) exposed to fulvestrant, Figure 4B. To elucidate the role of ERβ the receptor was knocked-down (KD) using siRNA. KD *per se* increased the proliferation rate and the effects of fulvestrant was diminished when ERβ expression was lost confirming the role of ERβ in mediating the effects of fulvestrant, Figure 4C. The role of ERβ in the control of proliferation was further supported by treatment with the selective ERβ antagonist PHTPP, which also resulted in a significant increase in the proliferation *per se* from 1±0.03 in untreated cells to 1.3±0.02 in the exposed cells, p<0.001, n=6 in each group.

### Moderate therapeutic effects of fulvestrant in triple negative weak ERβ+ MDA-MB-468 breast cancer explants

**Figure 5:**
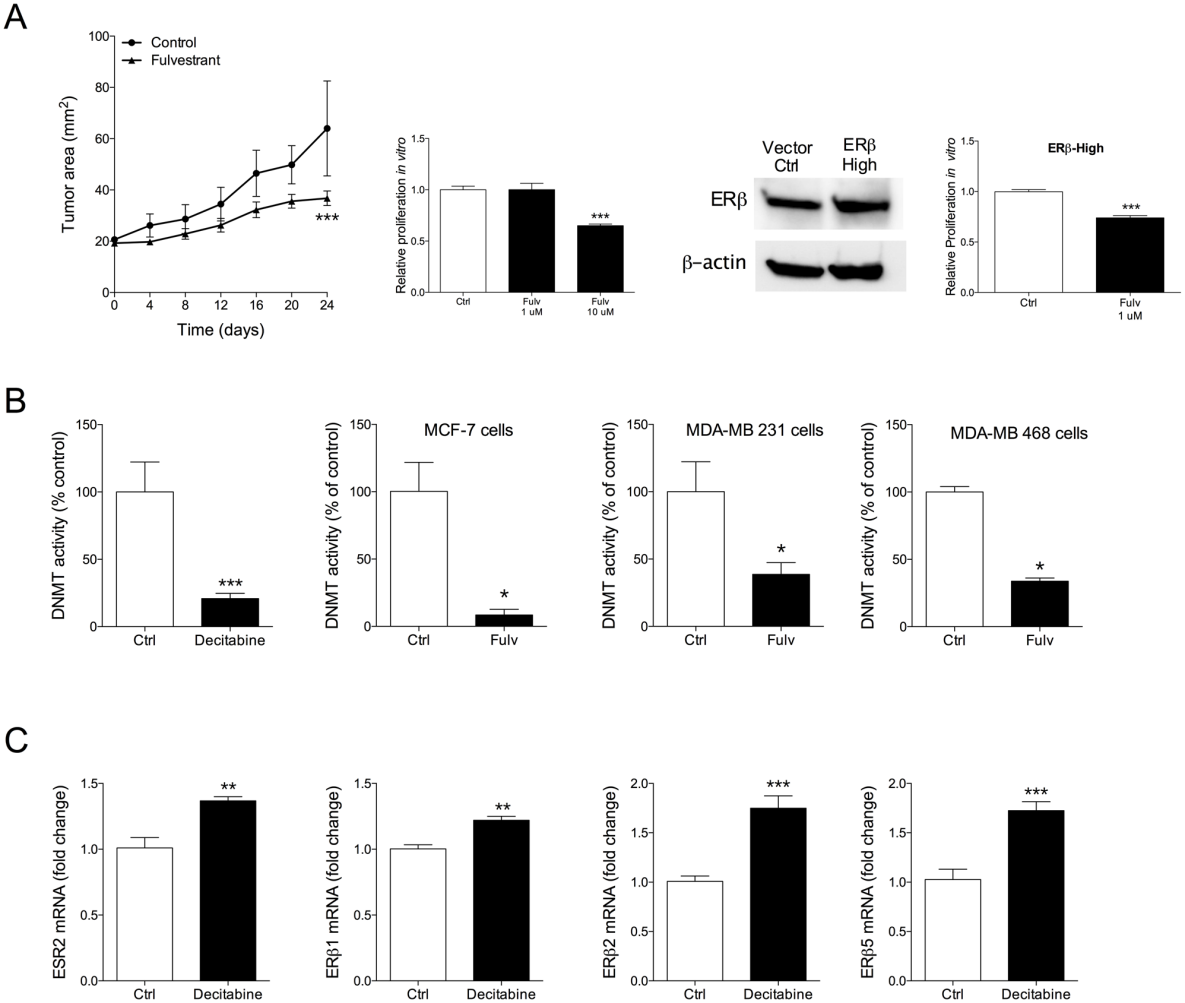
A. Moderate therapeutic effects of fulvestrant in triple negative weak ERβ+ MDA-MB-468 MDA-MB-468 (ERα-/weak ERβ+) cells were injected in the mammary fat pad in nude mice as described under figure 1. ***P<0.001 compared to control, n=6-8 in each group. Parental MDA-MB-468 or MDA-MB-468 where ERβ was knocked-in, confirmed with Western blot, were cultured *in vitro* and exposed to fulvestrant (Fulv) at different concentrations. Proliferation was measured, ***P<0.001 compared to control, n=6 in each group. Bars represent mean±SEM. **B.** Fulvestrant inhibited DNA methyltransferase activity (DNMT). DNA methyltransferase activity was measured in nuclear extract from the different cell lines exposed to fulvestrant. The DNMT inhibitor decitabine was used as a positive control. *P<0.05, ***P<0.001 compared to control, n=6 in each group. Bars represent mean±SEM. **C.** Inhibition of DNA methyltransferase activity increased ERβ and its isoform expression. Cells were exposed to decitabine and ERβ and its isoforms were analyzed at mRNA levels, **P<0.01 and ***P<0.001 compared to control, n=6 in each group. Bars represent mean±SEM.

To test our hypothesis that fulvestrant exerts its effects by ERβ and that the effects are depending on levels of ERβ expression we set up MDA-MB-468 tumors in nude mice. MDA-MB-468 cells do not express ERα and ERβ is express at lower levels compared with MDA-MB-231 cells. This was confirmed in our hands as we found that the relative expression of ESR2 was 4-fold higher in MDA-MB-231 cells (0.004±0.0004) than in MDA-MB-468 cells (0.001±0.0005). *In vivo*, fulvestrant treatment resulted in decreased tumor growth compared with controls but the effects were not as potent as in the MDA-MD-231 explants, Figure 5A. *In vitro*, the proliferation of MDA-MB-468 cells was not affected by fulvestrant exposure at 1 μM but at a higher dose (10μM) the cells decreased the proliferation rate, Figure 5A. The role of ERβ expression was further confirmed in cells where ERβ was increased; these cells responded to fulvestrant at lower concentrations and in a similar fashion as MDA-MB-231 cells, Figure 5A.

### Fulvestrant inhibited DNA methyltransferase activity

ERβ expression may be regulated by methylation of the promoter region of ERβ. This process is dependent on DNA methyltransferase (DNMT). Therefore, we examined if fulvestrant could inhibit DNMT. Indeed, we found that fulvestrant significantly decreased DNMT in all three cell lines in a similar fashion as the DNMT inhibitor decitabine, Figure 5B. Next, we investigated if DNMT inhibition *per se* affected the expression of ERβ. As shown in Figure 5C decitabine exposure resulted in increased expression of ERβ and its isoforms ERβ1, ERβ2, and ERβ5.

## DISCUSSION

Here we report that the pure anti-ERα drug fulvestrant increased ERβ expression both at mRNA and protein levels in ERα+/ERβ+ as well as in ERα-/ERβ+ breast cancers. In ERα+/ERβ+ breast cancer cells the increase of ERβ by fulvestrant was further enhanced in presence of tamoxifen. This led to an increased tumor regression when fulvestrant was combined with tamoxifen, both *in vivo* and *in vitro*, compared to either treatment alone. The decrease in tumor growth was accompanied with decreased proliferation and increased cell apoptosis. In ERα+/ERβ+ cells where ERβ was further increased by knock-in experiments the response to estrogen stimulation was decreased whereas the effects of fulvestrant increased supporting the role of ERβ in fulvestrant's therapeutic effects.

In ERα-/ERβ+ breast cancer fulvestrant exerted a therapeutic effect *in vivo* as well as *in vitro*. The effects were dependent on the levels of ERβ expression *per se* as triple negative cells with weak ERβ expression were less responsive to fulvestrant compared to triple negative cells with higher ERβ expression. In addition, when ERβ was knocked-in or knocked-down the response to fulvestrant changed accordingly. Inhibition of DNA methyltransferase by decitabine increased the expression of ERβ and its isoforms and fulvestrant inhibited DNA methyltransferase activities in all cell lines. Thus, effects on DNA methylation may be one of the mechanisms involved in fulvestrant's regulation of ERβ expression. In line with previous reports, fulvestrant degraded ERα, whereas tamoxifen increased ERα expression [29, 30].

Previously, it has been reported that ERβ inhibits breast cancer cell proliferation, migration, and invasion [31, 32]. In breast cancer cells where both ERα and ERβ are co-expressed ERβ may reduce ERα-mediated transcription by the formation of α/β heterodimers, with weaker transcriptional activity than the α/α homodimer or by a competition between ERα and ERβ homodimers in DNA binding, where α/α homodimers stimulate transcription more potently [33, 34]. Multiple ERβ isoforms exist as a result of alternative splicing or deletion of coding exons [16]. Several antibodies against ERβ directed to different isoforms of the receptor have been generated but the different antibodies do not exert similar staining patterns and there is no consensus of how to score tumor sections [16]. One of the isoforms, ERβ1, is the long form of the receptor and many functional studies have been derived by cloning of ERβ1 [35]. In clinical data sets loss of ERβ1 in breast cancers has been associated with poor survival [36, 37]. Moreover, the response to anti-estrogen therapy seems to be increased when ERβ1 is expressed [38]. Our results confirm these data as ERβ1 was among the different ERβ isoforms that were significantly increased with fulvestrant treatment. Regarding ERβ2, clinical studies have not yet reached any consensus regarding its role as a prognostic or predictive factor [16]. Moreover, different intracellular locations may also affect the prognostic significance of the different isoforms of ERβ [16]. Alteration in the expression of ERα/ERβ balance is a critical step in breast cancer development and progression, and selective restoration of the ratio is proposed as one of the major therapeutic approaches for breast cancer [37]. Our data support this, as up-regulation of ERβ *per se* increased the efficacy of fulvestrant and enhanced the effect with the combination therapy of fulvestrant and tamoxifen.

There are some discrepancies regarding the expression of ERβ in breast cancer cell lines in previous published studies [27]. Many studies have examined mRNA levels of ERβ alone without corresponding protein detection and there has been a lack of specific ERβ antibodies. Different antibodies are suitable in different conditions, and it is well known that members of the steroid receptor family are extremely labile proteins which may affect the degradation of specific epitopes during sample preparation [39]. In addition, the different splice variant of the receptor as well as antibodies raised against different epitopes may also affect the results. We confirmed that ERβ was expressed in all cell lines used in the present studies, both at mRNA and protein levels, and our results are in line with data from many other independent groups [40–50]. Moreover, the antibodies used in our study have been validated for each specific condition [49–52] and the specificity to ERβ was further confirmed in our study as the protein levels of the receptor increased or decreased when the receptor was knock-in or knocked down respectively.

The role of ERβ in breast cancers expressing ERβ alone, without ERα, is to date less clear and both increased and inhibited cell growth have been suggested [53, 54]. One reason for the different results may be presence or absence of a ligand to ERβ as it has been shown that up-regulation of ERβ induces a ligand independent decreased proliferation rate [53]. This is in line with our data showing that up-regulation of ERβ with fulvestrant decreased the proliferation rate without using any ligand in the experiments with the two triple negative cell lines. The level of ERβ expression affected the effect of fulvestrant as ERα- cells with intrinsically low ERβ expression exhibited less response to fulvestrant compared to ERα- cells with intrinsically higher ERβ expression. This was further highlighted when ERβ was up- or down-regulated as these cells changed their sensitivity to fulvestrant accordingly.

A few studies have suggested that tamoxifen may be beneficial for ERα- breast cancer [55, 56]. However, these patient materials were very small and they did not show similar results regarding the effects depending on menopausal status or HER-2 status [55, 56]. Another problem may be the lack of standardized staining protocol for ERβ. In the latest overview of all randomized trials investigating tamoxifen as an adjuvant it was concluded that ERα negative breast cancer have no benefit of tamoxifen therapy [57]. This is in line with our present data that do not support a therapeutic effect of tamoxifen on triple negative/ERβ+ breast cancer.

The expression of ERβ may be regulated by DNA methylation, a reaction that is catalyzed by DNA methyltransferase (DNMT) [58, 59]. Our present data suggest that fulvestrant is a potent inhibitor of DNMT as fulvestrant exhibited equal potency on DNMT activities as decitabine, a registered DNMT inhibitor. The role of DNMT in the regulation of ERβ was further demonstrated by our experiments using decitabine, which induced increased expression of ERβ and its isoforms in a similar fashion as fulvestrant. This suggests that an effect on DNMT activity is among the mechanism(s) involved in ERβ regulation by fulvestrant.

We conclude that fulvestrant is an ERβ targeted therapy leading to decreased breast cancer growth. In ERα+/ERβ+ this up-regulation resulted in enhanced therapeutic effects of the combined therapy of tamoxifen and fulvestrant compared to either treatment alone. As both of these drugs are currently in clinical use a clinical translation of our results may be feasible.

Our results in triple negative/ERβ+ models may also be translated into the clinical situation. TNBC is associated with worse clinical outcome with higher relapse rate and shorter overall survival compared with other sub-types of breast cancer [24]. The median survival time for women with metastatic TNBC is less than one year and the 5-year survival rate is considerable lower than other breast cancers despite adjuvant chemotherapy [23, 60].

The definition of TNBC is based on the lack of ERα. However, full length of ERβ protein has been detected in 30 to 90% of ERα negative breast cancers [56, 61, 62], and ERβ expression has been shown to correlate with improved disease-free survival and good prognosis in TNBC [56]. Thus, our results suggest that fulvestrant could be a potential ERβ targeted therapy in this group of breast cancer patients.

Consequently, we suggest that assessment of ERβ could be of value for several groups of breast cancer patients. Critical for further exploring ERβ and its variants, as diagnostic and prognostic tools and as a therapeutic target, is the development of standardized protocols for the detection of ERβ. However, an introduction of ERβ in clinical practice has the potential to improve clinical treatment decisions and the registered drug fulvestrant may be of benefit for new groups of breast cancer patients.

## MATERIALS AND METHODS

### Cells and culture conditions

MCF7 (ERα+/ERβ+), MDA-MB-231 (ERα-/ERβ+), and MDA-MB-468 (ERα-/ERβ+) were purchased from American Type Culture Collection; Manassas, USA. None of these cells express HER-2, thus MDA-MB-231 and MDA-MB-468 are defined as triple negative cell lines. Cells were maintained in phenol red-free DMEM with 2 mM glutamine, 50 IU/ml penicillin-G, 50 μg/ml streptomycin, 10% fetal bovine serum (FBS). All reagents were from Invitrogen (Carlsbad, USA) unless otherwise stated. Cells were treated with 10^−9^ M estradiol, 10^−6^ M tamoxifen, 10^−6^ M fulvestrant (Sigma-Aldrich, MO, USA) or in combination as indicated in DMEM/F12 (1:1) without phenol red supplement with 5% charcoal-stripped serum. The medium was changed daily. Dose response curves of fulvestrant were generated on the different cell lines, Supplementary Figure 1.

### Cell proliferation and cell cycle analyses

Cells were seeded at equal numbers 96-well plates and exposed to hormones as indicated and proliferation was measured using 5-bromo-2′-deoxyuridine (BrdU) proliferation kit according to the manufacturer's guidelines (BrdU cell proliferation ELISA, Roche, Mannheim, Germany).

For cell cycle analyses, cells were seeded at 100,000 cells/100 mm dishes and treated for 7 days, fixed in ethanol for 1 hour at 4°C, centrifuged and re-suspended in 1ml of propidium iodide staining solution (40μg/ml) (Sigma-Aldrich). 50μl of RNase A (10μg/ml) (Sigma-Aldrich) was added and the cells were incubated for 4 hours. Data was acquired on FACS Gallios (Beckman Coulter) and analyzed with ModFit LT (version 4, Verity Software).

### Quantitative polymerase chain reaction (qPCR)

Total RNA was extracted with Qiazol and RNeasy Mini kit (Qiagen, Maryland, USA). DNA was removed using DNase (Qiagen). RNA was converted to cDNA using High-Capacity cDNA Reverse Transcription kit (Applied Biosystems, CA, USA). Predesigned primers and probes (Applied Biosystems) or sequences adopted from published report were used [63]. Samples were pre-amplified for 14 cycles with TaqMan PreAmp mastermix (Applied Biosystems) for 10 min at 95°C and 14 cycles at 95°C for 15 second followed by 60°C for 4 minutes. Samples were diluted and qPCR was performed with TaqMan Fast Universal Mastermix (Applied Biosystems) and TaqMan gene expression assay on 7900HT Fast Real-Time PCR system (Applied Biosystems) using the amplification protocol; 95°C for 20 second, followed by 40 cycles of 95°C for 1 second and 60°C for 20 second. cDNA input was normalized with house-keeping gene (HPRT1).

### Protein detection of ERβ

For Western blot cells were lyzed in RIPA buffer containing protease inhibitor cocktail (Roche Diagnostics, Mannheim, Germany) and total protein concentration was measured using Pierce™BCA protein assay kit (Pierce Biotechnology, Rockford, USA). Equal amounts of protein, 100 μg, were separated using SDS/Page on mini-protean TGX gels (BioRad Laboratories, USA) and transferred to PVDF membranes (BioRad Laboratories, USA), which were incubated with mouse anti-human ERβ (Leica Biosystems, Newcastle, United Kingdom), rabbit anti-human β-actin (Cell Signaling Technology, Danvers, MA, United States) and horseradish peroxidase (HRP)- conjugated secondary antibodies from Dako (Dako, Glostrup, Denmark). Proteins were visualized using ECL detection reagent (Ge Healthcare, Buckinghamshire, UK) and ChemiDoc™Touch Imaging System and Image lab 5.2.1 analysis software from BioRad. A Western blot of ERβ expression of all cell lines and a negative control is shown in Supplementary Figure 3.

For quantification of ERβ an ELISA kit, Cat# E90437Hu (Wuhan USCN, TX, USA), was used.

### Animals studies

Mice were housed at Linköping University and the care and treatment conformed to the regulatory standards. The institutional animal ethics committee at Linköping University approved the study. Female athymic nude mice (Balbc nu/nu, 6–7 weeks old, Scanbur, Solna, Sweden) were housed in a pathogen-free isolation facility with a light/dark cycle of 12/12 hour, fed chow and water *ad libitum*. Mice were anaesthetized with intraperitoneal (i.p.) injections of ketamine/xylazine and oophorectomized, and implanted subcutaneously (s.c.) with 3-mm pellets containing 17β-estradiol, 0.18 mg/60-day release (Innovative Research of America, Sarasota, USA), resulting in serum concentrations of 150 to 250 pM [64, 65]. One week after surgery experiments were continued. MCF-7, MDA-MB-231, or MDA-MB-468 at 5 × 10^6^ cells in 200 μl PBS, were injected in the dorsal mammary fat pad. At a tumor size of approximately 20 mm^2^, mice were divided into subgroups; control, tamoxifen (1 mg/mouse every 2nd day s.c.) or fulvestrant (5 mg/mouse every 3rd day s.c.), and in the MCF-7 animals a combination of tamoxifen and fulvestrant.

### Immunohistochemistry

Formalin-fixed, paraffin-embedded tumors were cut in 4 μm sections, de-paraffinized, and exposed to mouse anti-human Ki67 (clone MIB-1, Dako), rabbit monoclonal anti-human ERα (clone SP1, Dako), two different mouse anti-human ERβ, (clone PPG5/10 ERβ1 and clone 57/3 ERβ2, AbD Serotec, Puchheim, Germany), rabbit anti-human cleaved PARP (Abcam, Cambridge, UK) or rabbit anti-human von Willebrand factor (Dako) followed by anti-rabbit or anti-mouse HRP polymer kit (Dako). Sections were counterstained with Mayer's hematoxylin. Negative controls with omitted primary antibodies were included in each experiment and did not show any staining. All evaluation was performed in a blinded manner. Images of hot-spot areas of at least four tumors in each treatment group were acquired on an Olympus BX43F microscope (Lund, Sweden). The images were digitally analyzed and quantified using Olympus CellSens Dimension software version 1.5 (Hamburg, Germany). Clone 57/3 ERβ2 is shown in Supplementary Figure 2.

### ERβ modulation

Parental MCF-7 and MDA-MB-468 were used to generate stable MCF-7/ERβ-high, MCF-7/ERβ-low, and MDA-MB-468/ERβ-high cells. MCF-7 cells were seeded at the density of 5 × 10^5^ cells per well in 6-well plates. Two different pre-designed human constructs in retroviral pGFP-V-RS vectors for ESR2 and scrambled negative controls non-effective shRNA cassette in pGFP-V-RS plasmid (Origene, MD, USA) were used. 1μg of plasmid DNA was mixed in 250μl of Opti-MEM 1 (Gibco) and 3μl of Turbofectin 8.0 (Origene) was added to the diluted plasmid DNA. Transfection was carried out in complete medium and ERβ low expressing cells were selected after growing the cells in medium containing puromycin. The shRNA with the most efficient inhibition of ESR2 was used for the experiment (fold decrease 0.5±0.01 vs 0.8±0.08). Decrease in ERβ expression after transfection was confirmed with qPCR and Western blot. Cells were transfected with pCDNA3.1nv5-ERβ (Addgene, Cambridge MA, USA). Clone was expanded and plasmids were extracted. 1μg of plasmid DNA was mixed in 250μl of Opti-MEM 1 (Gibco) and 3μl of Turbofectin 8.0 (Origene) was added to the diluted plasmid DNA. Transfection was carried out in complete medium. High-ERβ expressing cells were selected using neomycin containing growth medium. Increase in ERβ expression was confirmed with qPCR and Western blot.

For transient transfection, cells (5 × 10^5^) were plated in 6-well plates in phenol red-free DMEM media supplemented with 10% FBS. During transfection media was changed to hormone media. Transfection was performed on 60% confluent cells with Lipofectamine RNAiMax transfection reagent (Life Technologies) using either 25 × 10^−12^ M of either predesigned and validated ESR2 silencer select or negative control siRNA (Life Technologies). Opti-MEM (Life Technologies) was used during preparation and mixing of transfection agents. Forty-eight hours after transfections, ESR2 knock-down (KD) was validated with qPCR and Western blot.

### DNA methyltransferase activity

The effect of fulvestrant on DNA methyltransferases activity was measured by colorimeteric method (Abcam) in nuclear extract from cells. Decitabine (0.5uM, Tocris) was used as positive control.

### Statistical analyses

Student's t-test and ANOVA with Tukey's post hoc-test were used where appropriate. All experiments were carried out in multiple replicates. Graphpad Prism 6.0 was used for analyses (Graphpad Software, San Diego, USA). Data are expressed as mean ± SEM.

## SUPPLEMENTARY MATERIALS FIGURES


