# Supplementary material for: Fulvestrant inhibits growth of triple negative breast cancer and synergizes with tamoxifen in ERα positive breast cancer by up-regulation of ERβ
Source: Oncotarget. 2016 Jul 28;7(35):56876–88. doi: 10.18632/oncotarget.10871 (PMC5302959; doi:10.18632/oncotarget.10871)
Supplement: Supplementary file 1 [file oncotarget-07-56876-s001.pdf]

Fulvestrant inhibits growth of triple negative breast cancer and synergizes with tamoxifen in ERα positive breast cancer by up-regulation of ERβ

SUPPLEMENTARY DATA

The neurblastoma cell line SH-SY5Y (a genereous gift from Dr Hallbeck, Linköping University), which has been shown to be ERα positive and ERβ negative by several independent groups was used as negative control for validation of the ERβ antibody in a Western blot analysis [1-3].

REFERENCES

1. Wong J, Weickert CS. Transcriptional interaction of an estrogen receptor splice variant and ErbB4 suggests convergence in gene susceptibility pathways in schizophrenia. J Biol Chem. 2009; 284:18824-18832.
2. Shen B, Wang Y, Wang X, Du Y, Guo S, Cong L. Estrogen induced the expression of ADAM9 through estrogen receptor alpha but not estrogen receptor beta in cultured human neuronal cells. Gene. 2016; 576:823-827.
3. Su C, Rybalchenko N, Schreihofer DA, Singh M, Abbassi B, Cunningham RL. Cell Models for the Study of Sex Steroid Hormone Neurobiology. J Steroids Horm Sci. 2012; S2.

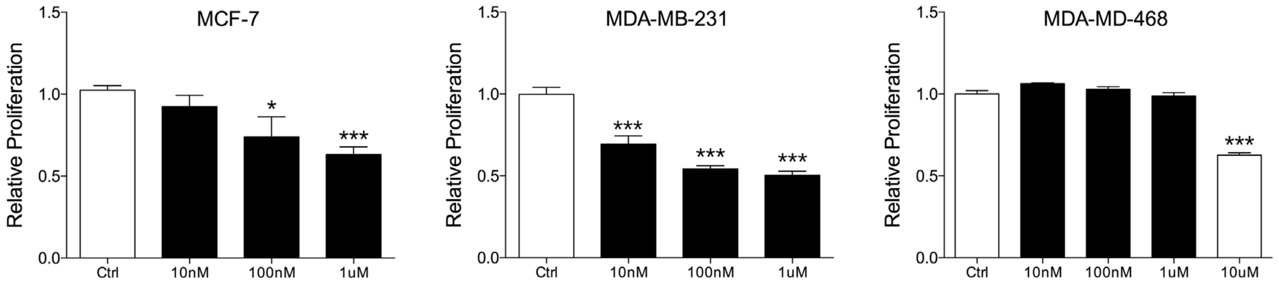

Supplementary Figure S1: MCF-7, MDA-MB-231, and MDA-MB-468 cells were cultured and exposed to fulvestrant at different concentrations. Proliferation was measured as described in the materials section. *P<0.05 and ***P<0.001 compared to controls, n=8 in each group. Bars represent mean±SEM. The control group represents the normalized absorbance of unexposed (hormone media alone) cells.

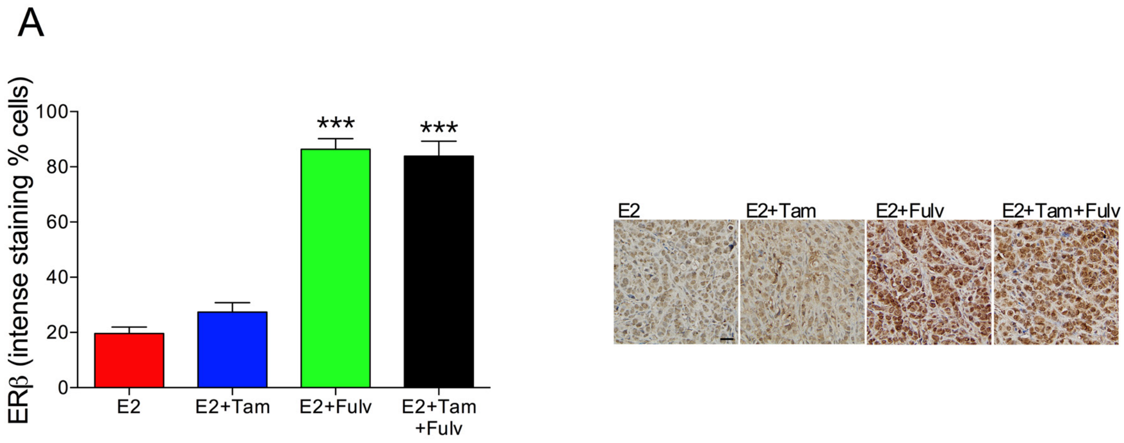

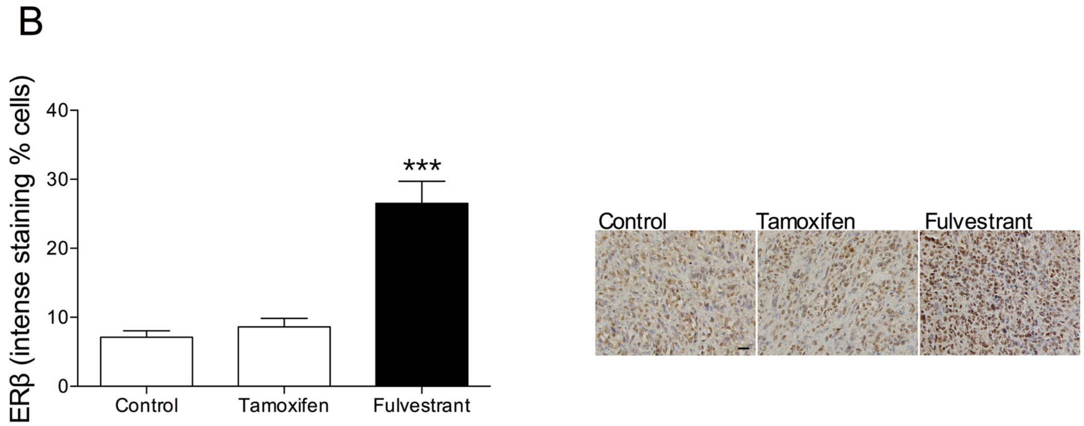

Supplementary Figure S2: Oophorectomized Balb/C-nu/nu mice were supplemented with physiological levels of estradiol (E2) and injected with MCF-7 or MDA-MB-231 cells in the mammary fat pad. Tumor sections from the different treatment groups were stained for ERβ (clone 57/3) and quantified as described in Materials and Methods. **A.** At similar tumor sizes, MCF-7 tumors were treated as follows; one group continued with E2 treatment and the other group received an additional tamoxifen (Tam) treatment (1 mg/mouse every second day s.c.), fulvestant (Fulv) (5mg/mouse twice weekly s.c.), or their combination. **B.** MDA-MB-231 tumors were treated as follows; one group were untreated, one group received tamoxifen treatment (1 mg/mouse every second day s.c.) and one group received fulvestant (5mg/mouse twice weekly s.c.). Representative sections from each treatment group are shown. Scale bars=50 µm. ***P<0.001 compared to the control group n=9 in each group. Bars represent mean±SEM.

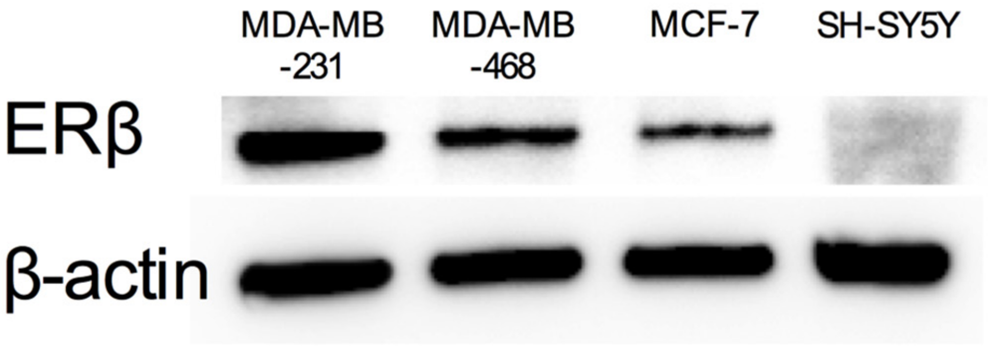

Supplementary Figure S3: Western blot for validation of the antibody and confirmation of protein expression of ERβ in the three different breast cancer cell lines MDA-MB-231, MDA-MB-468, MCF-7, and a negative control consisting of the neurblastoma cell line SH-SY5Y.
